# *Entamoeba histolytica* Calreticulin Induces the Expression of Cytokines in Peripheral Blood Mononuclear Cells Isolated From Patients With Amebic Liver Abscess

**DOI:** 10.3389/fcimb.2018.00358

**Published:** 2018-10-18

**Authors:** Enrique Gonzalez Rivas, Cecilia Ximenez, Miriam Enriqueta Nieves-Ramirez, Patricia Moran Silva, Oswaldo Partida-Rodríguez, Eric Hernandez Hernandez, Liliana Rojas Velázquez, Angelica Serrano Vázquez, Ulises Magaña Nuñez

**Affiliations:** Laboratorio de Inmunología, Unidad de Investigación de Medicina Experimental, Facultad de Medicina, Universidad Nacional Autónoma de México (UNAM), Ciudad de Mexico, Mexico

**Keywords:** amebic liver abscess, calreticulin, *Entamoeba histolytica*, mitogen, proliferation index, interleukins

## Abstract

Calreticulin (CRT) is a highly conserved protein in the endoplasmic reticulum that plays important roles in the regulation of key cellular functions. Little is known about the participation of *E. histolytica* CRT (*Eh*CRT) in the processes of pathogenicity or in the modulation of the host immune response. The aim of this study was to evaluate the role of CRT in the proliferation and the cytokine profile in peripheral blood mononuclear cells (PBMCs) from patients with amebic liver abscess (ALA) during the acute phase (AP-ALA) of the disease compared to patients during the resolution phase (R-ALA). The PBMCs from each participant were cocultured with *Eh*CRT and tested by the colorimetric method to evaluate their proliferation index (PI). The supernatants were subjected to an enzyme-linked immunosorbent assay (ELISA) to evaluate the concentration of cytokines. The mean values of all groups were compared using the independent *t*-test. When the PIs of individuals without diagnosis of liver abscess (NEG) were compared, there were no statistically significant differences in the proliferation of PBMCs between patients with AP-ALA and R-ALA when stimulated with *Eh*CRT or concanavalin A (ConA). However, the levels of interleukins [IL-6, IL-10, granulocyte colony stimulating factor (GCSF), and transforming growth factor β1 (TGFβ1)] were higher in patients with AP-ALA, whereas in patients with R-ALA, higher levels of interferon gamma (IFNγ) were detected. These results suggest that *Eh*CRT acts as a mitogen very similar to the activity of ConA. In addition, *Eh*CRT is an excellent immunogen for the specific activation of PBMCs, inducing the differential expression of ILs depending on the outcome of disease, determining the type of immune response: a Th2 cytokine profile during the acute phase and a Th1 profile during the resolution phase.

## Introduction

Infection with the enteric protozoan *E. histolytica* is one of the leading causes of death worldwide. The disease is a consequence of the parasite's abilities to invade the colon, causing amebic colitis. *E. histolytica* can disseminate to the liver via the portal venous system, resulting in amebic liver abscess (ALA). However, approximately 90% of infected people are asymptomatic cyst carriers (Haque et al., 2003). The molecular mechanisms by which this parasite causes invasive amebiasis are not fully understood. *E. histolytica* has adherence and cytotoxicity factors that are essential for its survival, but they are not directly responsible for ALA formation. It is known that the limitation and prevention of recurrent invasive amebiasis requires the development of an effective immune response. Thus, it is likely that the acute inflammatory response associated with *E. histolytica* infection is a key factor for the development of ALA (Chadee et al., 1985).

Parasite-specific immune responses are regulated by cytokines and chemokines that lead to the development of immunity, but these responses also contribute to infection, inducing pathogenesis and parasite persistence (Talvani et al., 2004). Little is known regarding the amebic signals that initiate an acute inflammatory response.

It has been reported that in mice infected with *E. histolytica*, host tissue damage is attributed primarily to the lectin activity of the galactose/*N*-acetyl-d-galactosamine (Gal/GalNAc) from *E. histolytica*, which promotes the accumulation of mononuclear cells, including neutrophils, inflammatory monocytes, and macrophages, at the site of infection (Blazquez et al., 2007). Intestinal epithelial cells infected with *E. histolytica in vitro* produce elevated levels of the cytokines, interleukin-8 (IL-8), growth-regulated protein alpha (GRO-α), granulocyte macrophage colony-stimulating factor (GMCSF), and IL-1 (Eckmann et al., 1995). Treatment of cultured human intestinal cells with the lectin Gal/GalNAc from pathogenic and nonpathogenic entamebas (*E. histolytica* and *E. dispar*) results in the secretion of chemoattractant and proinflammatory cytokines (Sharma et al., 2005), suggesting that these cells and cytokines also contribute to tissue damage, participating in the mechanisms of initiation, amplification, or limitation of the inflammatory processes during invasive amebiasis.

The identification of the mediators involved in leukocyte activation during infection by *E. histolytica* is of fundamental importance for understanding host responses in amebiasis. Cellular interactions and cytokines have been reported during amebic infections, and cytokines have been shown to be able to regulate monocyte function and increase the amebicidal activity of monocytes (Seydel et al., 2000; Lotter et al., 2013).

It is still not clear what other elements in the dynamics of host–parasite relationship define the outcome of infection, especially regarding the regulation of the immune response against *E. histolytica*.

To obtain further evidence about the relationships of immune cells with *E. histolytica*, other proteins have been studied to investigate the intracellular signals that promote the host immune response. Some examples include the cysteine proteinases 1 and 5 (CP1 and CP5) that breakdown IgA1 and IgA2 antibodies. These proteins cleave the Fc region that interacts with parasite surface receptors and mediates effector functions that can mask immunogenic surface molecules with inert Fab fragments, thus helping to prevent the parasite expulsion from the intestinal lumen (Garcia-Nieto et al., 2008).

The role of calreticulin (CRT) in host–parasite interactions has recently become a major area of research. The CRT genes from many parasites (*Trypanosoma, Leishmania, Entamoeba, Onchocerca, Schistosoma*, and *Haemonchus*) have been cloned and sequenced (Rokeach et al., 1994; Joshi et al., 1996; El-Gengehi et al., 2000; Marcelain et al., 2000; González et al., 2002; Suchitra and Joshi, 2005).

Although the functions of CRT are conserved in vertebrates, some CRT functions differ among parasites (Nakashi et al., 1998; Ferreira et al., 2004); parasite CRTs bind host C1q and inhibit C1q-dependent complement activation. The CRT of *Haemonchus contortus* binds host C-reactive protein and C1q (Naresha et al., 2009). The ecto-parasite *Amblyomma americanum* secretes CRT during feeding, suggesting that the anticoagulant ability of CRT may prevent blood clotting and allows the parasite to feed on the host and induce host antiparasite responses (Jaworski et al., 1995). The presence of CRT in the penetration gland cells of *Schistosoma* suggests that this molecule may be important for the host skin penetration (Khalife et al., 1994).

Previously, we reported the presence of CRT in *E. histolytica* (*Eh*CRT) and that this protein induces an important immunogenic response in the human host. More than 90% of patients with ALA develop high levels of serum antibodies against *Eh*CRT (González et al., 2002). We also reported the cloning of the CRT gene from *E. histolytica*, and the preparation of mono-specific antibodies against recombinant CRT. The immunohistochemical assays on trophozoites show that *Eh*CRT is in cytoplasmic vesicles and in vesicles that are in close contact with the inner cytoplasmic membrane (González et al., 2011). In addition, it was demonstrated that the CRT from both pathogenic *E. histolytica* and nonpathogenic *E. dispar* species specifically interact with human C1q molecules and inhibit the activation of the classical complement pathway (Ximénez et al., 2014). This activity is consistent with that reported by Vaithilingam et al. (2012). The trophozoites activated by the presence of jurkat cells clearly show the binding of C1q to CRT on the surface of the phagocytic mouths during the process of erythrophagocytosis.

However, the activation of the host immune response and the cytokine profile induced by *Eh*CRT have not yet been investigated.

In this study, we analyzed the proliferation and cytokine production of peripheral blood mononuclear cells (PBMCs) cultures isolated from ALA patients during the acute phase of the disease and from individuals who resolved ALA in order to characterize the cytokines profiles produced in response to *Eh*CRT.

## Methods

### Ethics statement

The present work was designed according to the guidelines for the management of human samples for experimental purposes as indicated in the Official Regulation NOM-12SSA3-2007 included in the General Health Law of Mexican Health Ministry. In addition, the project was approved by the Scientific and Ethics Committee of the Faculty of Medicine from the National Autonomous University of Mexico. Patients were informed about the purposes of the project, the sampling, and the potential risks during procedure, and the patients were invited to voluntarily participate by signing an informed consent letter.

### Study groups and biological samples

Patients with diagnosis of ALA admitted to the Internal Medicine, Gastroenterology, and Infectiology Services of the General Hospital of Mexico Dr. Eduaro Liciaga were recruited. These patients formed the group with acute phase amebic liver abscess (AP-ALA). The patients in the resolution phase of ALA (R-ALA) were recruited from a search of the archives of discharged patients from the General Hospital of Mexico, and they formed the ALA resolution group (R-ALA). Three fecal samples were collected from each patient from both groups at 1-week intervals after their hospitalization for microscopic examination for parasites. At the time of collection of the third sample, 10 ml of blood was drawn to evaluate serum antibodies against *E. histolytica* by enzyme-linked immunosorbent assay (ELISA), using an OD_490nm_ ≥0.5 as an indicator of a positive result (Morán et al., 2007). The remaining sample was used to isolate PBMCs.

A third group, named the negative control (NEG), was formed with clinically healthy individuals from the Blood Bank of the General Hospital of Mexico, with ELISA serum levels of antiamebic antibodies below the OD threshold (OD < 0.5). Using the same protocol for ALA groups, three fecal samples and 10-ml blood samples were taken for microscopic examination and PBMC isolation, respectively. Ten individuals for the group were included.

### Isolation and culture of PBMC

The PBMCs were isolated from 10 ml peripheral blood samples collected from each participant in tubes with K2 ethylenediaminetetraacetic acid (EDTA) as anticoagulant (BD Vacutainer, Ref 368171). Cells were separated on a Ficoll-Hypaque gradient (Gibco, Life Technologies, Grand Island, NY, USA), and the PBMC pellet was separated and washed three times with phosphate-buffered saline PBS. After separation, PBMCs were centrifuged and resuspended in Roswell Park Memorial Institute medium RPMI culture medium supplemented with 10% fetal bovine serum. The cells (1 × 10^6^ cells/ml) were incubated with r*Eh*CRT (5 μg/ml) at 37°C with 5% CO_2_; concanavalin A (ConA) (Sigma Chemical St. Louis, MO USA) was used as a stimulating factor at 10 μg/ml for different periods of time (24, 48, 72, and 96 h). Each experiment was performed in duplicate. At each time point, the cultures were centrifuged for 10 min at 1,000 × g, and the supernatant was reserved for cytokine analysis.

### Recombinant *Eh*CRT production

Full-length r*Eh*CRT protein was expressed and purified as previously described (González et al., 2011). Briefly, the plasmid pBluescript-KS+ (pbKS+) was used to clone and express the 1,178 bp *Eh*crt gene (GB-EAL649855.1) (Loftus et al., 2005), which produces the full-length protein; it was subcloned into the prokaryotic expression vector pProEX HT-b (Gibco Life Technologies) to express the CRT protein with a six-histidine tag at the N-terminal end. Competent *Escherichia coli* BL21 cells were transformed with the recombinant plasmid. The expression of recombinant protein r*Eh*CRT was induced with a final concentration of 1 mM isopropyl-β-D-thiogalactoside. The QIAexpressionist system (Qiagen, Valencia, CA, USA) was used to purify the recombinant protein, and, briefly, the cells were harvested by centrifugation at 3,000 × g for 12 min, and the bacterial pellet was resuspended in 5 ml lysis buffer (8 M urea, 0.1 M NaH_2_PO_4_, and 0.1 M Tris-HCl, pH 8.0). The lysate was added to a 50% suspension of Ni-NTA agarose (Qiagen,). The mixture was passed through a filtration column, and the recombinant protein was eluted with 8 M-urea buffer pH 4.5. The selected fractions were dialyzed against 19 mM PBS. Protein concentration was determined by the Bradford method, and the quality was evaluated by 12% sodium dodecyl sulfate polyacrylamide gel electrophoresis (SDS–PAGE). The reactivity grade of the recombinant protein was tested against sera from patients with ALA and for anti-*E. coli* lipopolysaccharide antibody (LPS) antibody (ab211144, Abcam) by Western blot.

### Proliferation index (PI)

To obtain the PI at 24, 48, 72, and 96 h, 20 μl of 5 μg/ml [3-(4,5-dimethylthiazol-2-yl)-2,5-diphenyltetrazolium bromide] (MTT) (Sigma) was added to each well containing PBMCs incubated with r*Eh*CRT or ConA and incubated at 37°C for 1 h in a humidified incubator with 5% CO_2_. After incubation, the plates were centrifuged at 1,000 × g for 10 min and the supernatant was discarded. The cells were resuspended in 300 μl dimethyl sulfoxide (Sigma) and the optical density (OD) was measured at 570 nm in an ELISA plate reader (EL × 800 BioTek). The proliferation index was calculated by [OD of test sample—OD of negative control/OD of negative control]. (Verma et al., 2010)

### Cytokine detection by ELISA

The supernatants of the PBMC cultures, treated with r*Eh*CRT, ConA, or without stimulus (RPMI-10% SFB), were tested for detection of interleukins IL-2, IL-4, IL-5, IL-6, IL-10, IL-12, IL-13, IL-17A, interferon gamma (IFNγ), tumor necrosis factor alpha (TNFα), granulocyte colony stimulating factor (GCSF), and transforming growth factor beta1 (TGFβ1) using a multianalytic ELISA array kit (MEH-003A, Qiagen), according to manufacturer's instructions. The concentrations of the cytokines are given in pg/ml and were calculated using the standard curve provided in the kit.

### Statistical analysis

All values are expressed as the means ± S.D. The student's *t* test for unpaired results was used for the evaluation of differences between cytokine concentrations in each group. Differences were statistically significant when *P* ≤ 0.05.

## Results

### Study participants and *E. histolytica* antibody concentration

The demographic features of each group are shown in Table 1. Among all the individuals, the mean age was 39 ± 7 years old, and 70% were male and 30% were female. The microscopic examinations were negative in all samples. Results of ELISA assay are shown as OD _490nm_ values, considering negative results when values were under the cut value of 0.520. The mean value for the negative group was 0.15, the AP-ALA group was 0.87, and the R-ALA group was 1.0.

**Table 1:** Demographic characteristics of individuals in the AP-ALA, R-ALA, and healthy control (NEG) groups.

**Groups**	**Age (range in years)**	**Male**	**Female**	**Total**	**ELISA (OD)**
AP-ALA	39.11	9	1	10	0.87 ± 0.043
R-ALA	39.72	6	4	10	1.0 ± 0.025
NEG	47.62	7	3	10	0.15 ± 0.024

*The antibodies against E. histolytica antigens present in all belonging to different studied groups were evaluated by ELISA. The data are expressed as the average OD ± SD in each group*.

### Purification of r*Eh*CRT and PI

**Figure 1:**
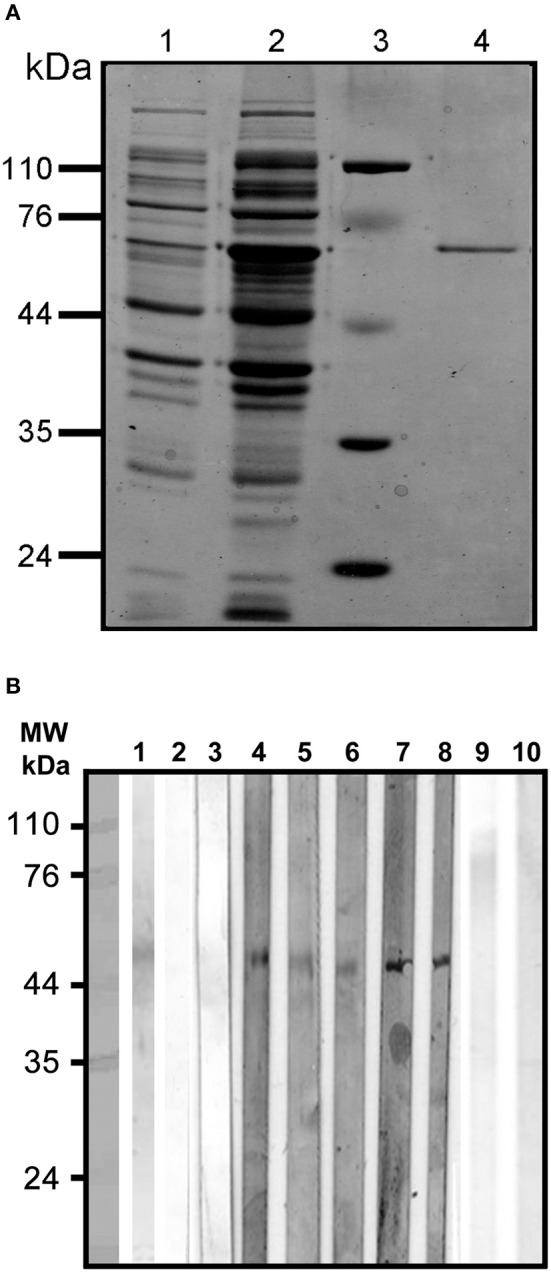
**(A)** Purification under denaturing conditions of rEhCRT. Proteins were visualized by 12% SDS-PAGE and Coomassie staining; (1) cell lysate with plasmid, (2) cell lysate with plasmid after IPTG induction, (3) molecular weight, (4) rEhCRT eluid from Ni-NTA Agarose column, molecular weight marker are indicated in kilodaltons. **(B)** Westernblot of rEhCR. The reactivity of the rEhCRT was tested against sera from the different groups studied; (1) 5 μg of rEhCRT electrotransfered to NC membrane, (2–3) serum from control individuals (NEG), (4–6) serum of AP-ALA patients, (7–8) R-ALA patients, and (9–10) reactivity against *E. coli*-LPS antibody.

The purified r*Eh*CRT from *E. coli* appeared as a single band at 60 kDa on 12% SDS-PAGE after bromophenol blue staining (Figure 1A). The results indicate that r*Eh*CRT was a good immunogenic factor, and this was previously confirmed by the antibody production in animal models (rabbits and mice) (González et al., 2011). The r*Eh*CRT conserved its reactivity when it was tested as an antigen using serum from ALA patients in ELISA assays (Table 1) and in Western blots, and no reactivity was observed with anti-*E. coli* LPS antibody (Figure 1B).

**Figure 2:**
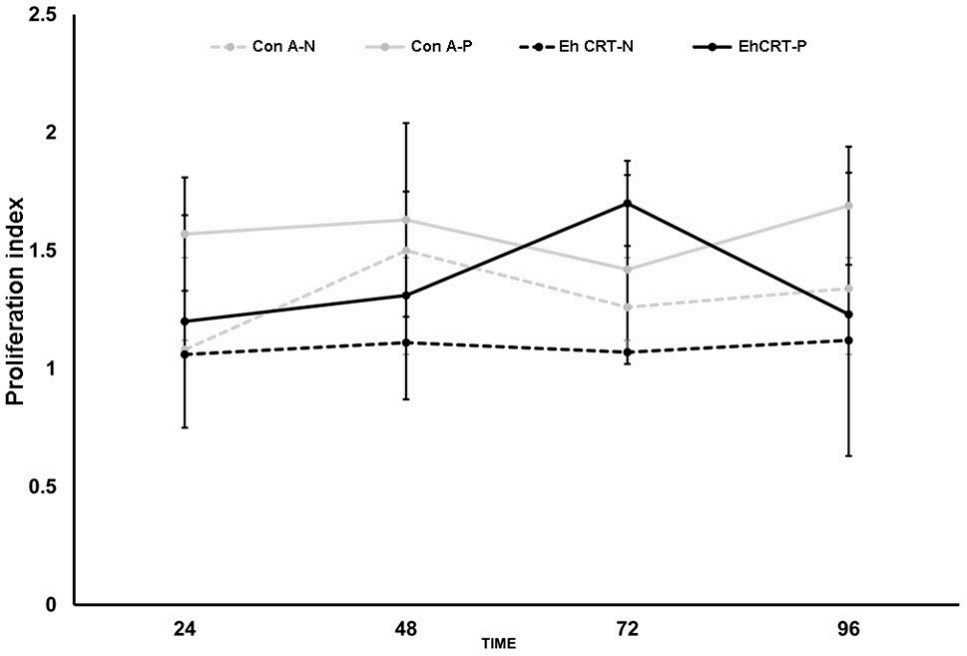
Proliferation Index at different times, of PBMC cells obtained from control subjects (NEG) and ALA patients stimulated with rEhCRT or Con A.

**Figure 3:**
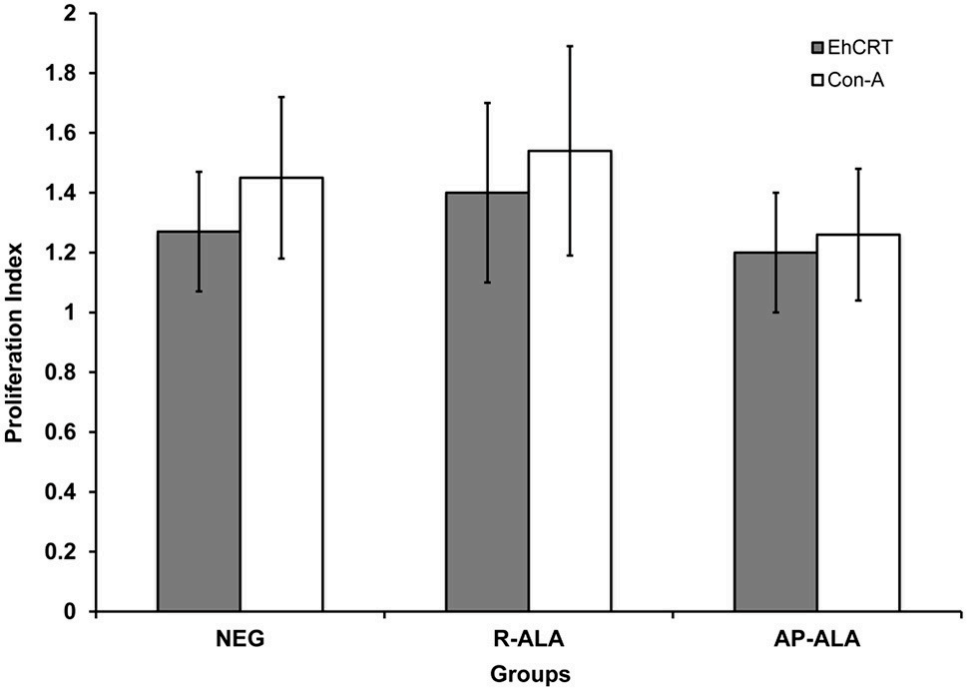
Proliferation Index at 72 h post incubation of PBMC cells obtained from control subjects (NEG), AP-ALA or R-ALA patients with rEhCRT or Con A. PI ≥1 is considered as positive stimulus.

The role of r*Eh*CRT as a costimulatory factor in the proliferation of PBMC was verified when cellular proliferation was measured by 3-[4,5-dimethylthiazol-2-yl]-2,5 diphenyl tetrazolium bromide (MTT) assays, comparing the stimulatory capacity of r*Eh*CRT vs. the mitogen activity of ConA. The differences in the PI values were not statistically significant for any time point when comparing between the NEG and ALA groups (AP-ALA, R-ALA) (Figure 2). However, at 72 h, we can see the highest PI (Figure 3) and the PI ≥ 1 is considered as positive proliferation (Verma et al., 2010).

### Detection of cytokines in the supernatants of PBMC cultures

**Figure 4:**
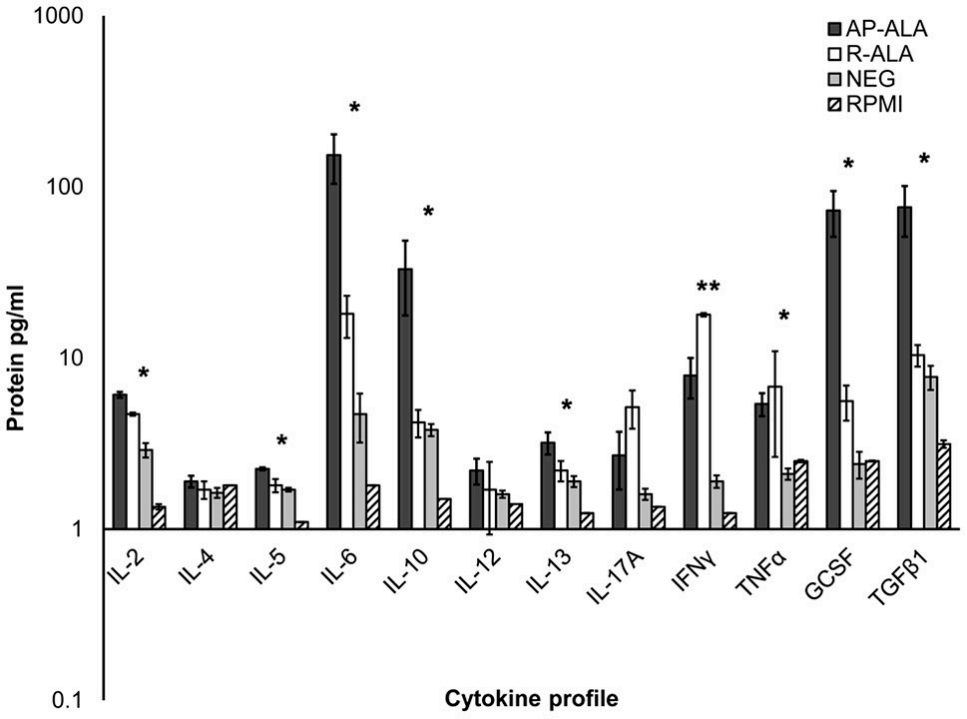
Cytokine profile on PBMC cells stimulated with rEhCRT. The data shows the concentration of cytokines, obtained in the supernatant of PBMC cells of different studied groups (NEG, AP-ALA, and R-ALA) after 72 h of incubation and stimulated with rEhCRT or without stimulus (RPMI). The concentration of interleukins is expressed as pg/ml in a logarithmic scale. *p ≤ 0.005, when compared groups of individuals (NEG) against the group of patients (ALA), **when compared AP-ALA against R-ALA groups.

The concentrations of cytokines in PBMC samples obtained for sera from the ALA and NEG groups and treated with EhCRT were measured through ELISA only at 72 h (Figure 4). The pattern of pro-inflammatory cytokines from the AP-ALA and R-ALA groups were compared to the NEG group, who did not produce the mentioned cytokines in the absenceof EhCRT.

The trend of the data agrees with an overexpression of all interleukins analyzed in all groups.

However, in the ALA groups, the overexpression was, in general, larger than in the negative group, and significant differences were measured in the expression of the following interleukins; IL-2, IL-5, IL-6, IL-10, IL-17, IFNγ, TNFα, GCSF, and TGFβ1 (*p* = 0.0035, 0.0023, 0.019, 0.006, 0.0076, 0.0049, 0.0039, 0.0035, 0.0059, respectively).

The comparisons of differential expressions of the interleukins between groups of AP-ALA and R-ALA are shown in Figure 4. We found differences in the overexpression of interleukins IL-2, IL-5, IL-6, IL-10, GCSF, and TGFβ1 (*p* = 0.0067, 0.026, 0.0045, 0.0016, 0.0051, 0.0047, respectively), which were higher in the AP-ALA group. In the R-ALA group, we observed higher overexpression of the interleukins IL-17, INFγ, and TNFα; however, statistically significant differences were detected only for INFγ (*p* = 0.73, 0.0014, 0.096).

## Discussion

The aim of this study was to examine the proliferation of PBMCs obtained from the blood of patients with AP-ALA and R-ALA stimulated *in vitro* with r*Eh*CRT or ConA and determine the cytokine profiles induced in the different groups. The responses of the PBMC show that *Eh*CRT is one of the many immunogenic proteins in *E. histolytica* that can induce activation and proliferation of PBMCs similarly to ConA. These results reinforce our previous observations that the *Eh*CRT is highly immunogenic in humans, mice, and rabbits (González et al., 2002, 2011).

When comparing the cytokine profiles between the AP-ALA and R-ALA groups in contrast with negative or PBMC without stimulus, it is clear that *Eh*CRT functions as a specific antigenic costimulator, inducing a different pattern of cytokines between different groups. This stimulatory action was specific because no reactivity with *E. coli*-LPS antibody was detected for the recombinant protein EhCRT (Figure 1A).

The expression levels of IL-2, IL-5, IL-6, IL-10, GCSF, and TGFβ1 were increased in patients with AP-ALA, while the expression levels of IL-17, INFγ, and TNFα were mainly upregulated in the R-ALA group. Nevertheless, there were statistically significant differences only for INFγ.

*E. histolytica* has different proteins that modulate the host immune response. The Gal/GalNAc-lectin induces the T cells to production of IL-2 and INFγ (Schain et al., 1992), whereas in macrophages, the amebicide activity of Gal/GalNAc-lectin induces the production of TNFα (Seguin et al., 1997). In dendritic cells, Gal/GalNAc-lectin favors a Th1 response in addition to inducing the production of major histocompatibility complex (MHC) class II molecules, and the costimulatory molecules CD80, CD86, and CD40 (Ivory and Chadde, 2007).

Another protein called monocyte locomotion inhibitory factor (MLIF) is produced by *E. histolytica* in axenic cultures that induces the production of pro-inflammatory cytokines (IL-1β, IL-2, IL-5, IL-6, IFN-γ) and anti-inflammatory cytokines such as IL-10, as well as the low expression of chemokines CCL1, CCL4, and the receptor CCR1 in human monocytes (Rico et al., 2003; Utrera-Barillas et al., 2003).

In the group of patients with AP-ALA, the cytokines IL2, IL5, Il6, IL17A, IFNγ, and TNFα displayed an increase in their concentration and demonstrated that the immune response had a pro-inflammatory profile. This response has been observed in stimulation assays using Gal/GalNAc-lectin in intestinal cell cultures (Sharma et al., 2005) and in other parasitic diseases such as malaria (Vazquez et al., 2015). It is important to highlight the effect on IL2, IL6, IL-17, IFNγ, and TNFα, whose levels were the highest and overexpressed in comparison with the profile observed in the negative group.

Guo et al. (2011), demonstrated in an *Entamoeba histolytica* vaccination model that IL-17 provides protection to mice vaccinated with the recombinant LecA fragment of the Gal/GalNAc-lectin. Interestingly, the major source of IL-17 in these mice was the CD8 T cells, whereas CD4 T cells express elevated levels of IFN-γ. The authors suggest that IL-17 may enhance the protective functions of Th1 immune response.

These results lead us to propose that treatment of PBMC in culture with *Eh*CRT favors the production of IFNγ and increases the production of IL-17A, thus directing the cellular immune response to a Th1 Profile, in PMBCs obtained from R-ALA.

Our results agree with those published by Ghadirian and Denis (1992), who showed that IFNγ could activate mouse peritoneal macrophages, which, in turn, were able to eradicate the *E. histolytica* trophozoites from colon tissue *in vitro*. Studies in animal models (Seydel et al., 2000) and human infections (Haque et al., 2007) have established that amoeba-specific IFN-γ production is critically involved in the clearance of infection and in host protection. In addition, Meza et al. (2014) demonstrated that virgin T-cell differentiation into Th17 cells producing IL-17 occurred after the direct stimulation with other cytokines such as TGFβ, IL-6, and IL-1 in a murine model of infection with *Trypanosoma cruzi*. Moraes et al. (2015) reported that mononuclear cells collected from healthy individuals incubated with *E. histolytica* in culture induced the production of *IFN*γ and TGFβ, and that both had a beneficial effect on the modulation of the activity of these cells. Our results agree with data of Moraes regarding the effects on IFNγ. These cytokines are important in the control *E. histolytica* infection.

The R-ALA group showed an increase in the concentration of the cytokines IL-10 and TGFβ, which agrees with results of Bansal et al. (2005). These authors mention that, in addition to the production of these cytokines and the increased production of IL-4, a suppressive immune response was also induced in patients infected with *E. histolytica*, which, in turn, favored a symptomatic infection. The symptomatic group in Bansal et al. differs with our R-ALA group because the latter had no symptoms and they were all ALA with negative microscopic examinations and a resolved *E. histolytica* infection. In our opinion, this immunosuppressive effect is due to the direct stimulation of the PBMC by *Eh*CRT, and through autocrine, paracrine, and pleiotropic effects on cytokine production, which favored the increase in other types of cytokines such as IL-5, IL-6, and IL-13 capable of generating a Th2 immune response.

In addition, we found that the increased cytokine IL-10 in the AP-ALA group in our study was in contrast with the results reported by Bansal. These results suggest that IL-10 is an immunomodulator resulting in proinflammatory cytokine profiles that could promote immunosuppression in the R-ALA individuals. The effect attributed to IL-10 differs in other parasitic diseases such as *Leishmania donovani* (Andreani et al., 2015), *Trypanosoma cruzi* (Longhi et al., 2014), and *Giardia duodenalis* (Babaei et al., 2016). In these reports, a decrease in IL-10 was observed that favored the spread of parasites into the hepatic tissue.

On the contrary, the IL-10 level was increased in dysenteric and ALA patients (Utrera-Barillas et al., 2003; Bansal et al., 2005). These studies indicate that invasion of the colon and liver by *E. histolytica* elicits an anti-inflammatory immune response and may successfully suppress immune reaction to the amebae.

In summary, the ameba needs to balance IL-10 and the pro-inflammatory cytokine to allow establishment of infection. In contrast, peritoneal monocytes and macrophages exposed to lipopeptidophosphoglycan (LPPG) secreted TNF-α, IL-6, IL-8, IL-12, and IL-10 via TLR2 (Maldonaldo-Bernal et al., 2005). Thus, LPPG-driven signaling may activate a negative feedback loop that attenuates inflammatory responses.

Host protective immunity involves participation of both humoral and cellular responses; however, the mechanism involved in the immune evasion of *E. histolytica* is not clear. One of these mechanisms could be associated with the ability of parasites to modulate cytokine expression in the inflammatory process, which is initiated by expression of proinflammatory cytokines. *E. histolytica* infections induce a state of transient suppression of cell-mediated immunity in early stages of inflammation in amebic hepatic abscess, and a complex signaling system of cytokines is triggered by pathogen invasion (Eckmann et al., 1993; Romagnani, 2000).

## Conclusions

The data obtained in this study confirmed that the *Eh*CRT behaves like an amebic immunogenic protein for humans and suggest that the *Eh*CRT participates in the specific stimulation of immune cells.

Our results suggest that the r*Eh*CRT can stimulate human PBMC proliferation independently of the presence of *E. histolytica* trophozoites, acting as a specific costimulator of the immune response like that induced by ConA. In addition, these results underline *Eh*CRT as a parasitic factor that can modulate the immune response, from the stimulation of proinflammatory cytokines to the immunosuppressive effects, depending on the progression of the ALA, thus inducing the development of a Th2 cytokine profile in the acute phase of disease and a Th1 profile once the individuals had resolved the ALA.

The functions of *Eh*CRT and its role in the pathogenesis of ALA need further research, particularly on the interaction with the cells of immune system and the induction of chemokines and cytokines regulators that hopefully will allow a better understanding of the pathogenesis of ALA.

## Author contributions

CX conceptualized the manuscript. EG, MN-R, UM. PM, OP-R, EH, LR, and AS curated the data. CX, EG, MN-R, PM, OP-R, EH, LR, and AS performed the formal analysis. CX acquired the funding. EG, MN-R, and PM performed the investigation. MN-R, PM, AS, and UM provided the resources. EG, OP-R, and EH performed the software analysis. CX, EG, MN-R, and PM provided the supervision. CX, EG, MN-R, and PM executed the validation. CX, EG, MN-R, and PM wrote the original draft of the manuscript. CX, EG, MN-R, PM, OP-R, EH, UM, and AS wrote, reviewed and edited the manuscript.

### Conflict of interest statement

The authors declare that the research was conducted in the absence of any commercial or financial relationships that could be construed as a potential conflict of interest.
